# Efficacy and safety of ultrasound-guided pulsed radiofrequency for cervicogenic headache: a retrospective study focusing on the C2 dorsal root ganglion at the C1–2 level

**DOI:** 10.22514/jofph.2025.010

**Published:** 2025-03-12

**Authors:** Xing Jin, Chenxi Li, Qingyu Zhang, Ye Sun, Xiangzheng Qin, Zhiting Dong, Wenzhe Jin, Kai Li

**Affiliations:** ^1^Department of Pain, Yanbian University Hospital, 133000 Yanji, Jilin, China; ^2^Department of Oral and Maxillofacial Oncology & Surgery, School/Hospital of Stomatology, The First Affiliated Hospital of Xinjiang Medical University, 830054 Urumqi, Xinjiang Uygur Autonomous Region, China; ^3^Clinical Medicine Postdoctoral Scientific Research Station, Xinjiang Medical University, 830054 Urumqi, Xinjiang Uygur Autonomous Region, China; ^4^Department of Anatomy, School of Medicine, Yanbian University, 133000 Yanji, Jilin, China; ^5^Department of General Surgery II, Affiliated Hospital of Beihua University, 132000 Jilin, Jilin, China; ^6^Department of Anesthesiology, China-Japan Union Hospital of Jilin University, 130021 Changchun, Jilin, China

**Keywords:** Ultrasound guidance, C2 dorsal root ganglion, Pulsed radiofrequency, Cervicogenic headache

## Abstract

**Background**: This study evaluated the effectiveness and safety of ultrasound-guided pulsed radiofrequency (PRF) at the C2 dorsal root ganglion (DRG), specifically at the C1–2 level, for patients with cervicogenic headaches. **Methods**: The study involved 29 patients with unilateral symptoms from January to July 2023. Headache intensity was measured using the numerical rating scale (NRS), with scores recorded before and after the procedure at specified intervals extending up to 24 weeks. Additionally, the neck disability index (NDI) scores were assessed at baseline, 4, 12 and 24 weeks. **Results**: The findings demonstrated significantly reduced headache NRS scores at all post-treatment checkpoints, with notable pain relief rates of 13.79% and 72.41% at 4 weeks, and 17.24% and 68.97% at 12 and 24 weeks, respectively. NDI scores also showed significant reductions at all evaluated post-treatment time points. Importantly, no significant adverse events were observed in any of the individuals. **Conclusions**: Our ultrasound-guided approach could be a safe and effective alternative for managing cervicogenic headaches.

## 1. Introduction

Cervicogenic headache (CEH) is a common type of secondary headache that is often linked to pathological injury of the cervical spine or restricted neck movement in patients [1]. The onset of this headache type is closely associated to anatomical abnormalities in the neck [1]. Research has indicated that spinal neurons in the upper cervical segment (C1–C3) can cause discomfort in the neck, but also in areas including occiput, crown of the head, auriculotemporal region, and periorbital and frontal areas when these neurons are subjected to mechanical compression and inflammation-induced irritation [2]. The estimated prevalence of CEH in the general population is approximately 4.1% [3] with CEH contributing to up to 17.5% of cases among individuals experiencing severe headaches [4].

The International Headache Society (IHS) initially introduced the International Classification of Headache Disorders (ICHD) in 1988. However, the diagnostic criteria for CEH were not definitely established until the publication of the second edition in 2004 [5]. The third version, ICHD-3, released in 2018, further refined and expanded the diagnostic criteria for CEH, providing comprehensive descriptions of headache symptoms and including requirements for imaging evidence [1]. The flexion-rotation test of the cervical spine, a clinical instrument for evaluating CEH, was also incorporated into the ICHD-3 as a supportive diagnostic method [5]. This test assists doctors in diagnosing and confirming CEH by evaluating the relationship between headache symptoms and cervical spine abnormalities through specific neck motions.

The management of CEH includes several methods, such as oral pharmacotherapy, physiotherapy, manual therapy, and acupuncture [6]. If these treatments are inadequate, minimally invasive procedures targeting the dorsal root ganglion (DRG) of the C2 nerve, such as glucocorticosteroid injections, local anesthetics, and radiofrequency therapy, are commonly utilized [5]. Pulsed radiofrequency (PRF) produces an oscillating electromagnetic field in the C2 DRG, which subsequently modifies neuronal potentials, regulates calcium ion channels, suppresses the release of inflammatory mediators, and influences gene expression. These mechanisms inhibit pain signal transmission, alter neuronal activity, reduce inflammatory responses, and result in long-lasting changes in pain pathways, ultimately offering effective therapies for neuropathic pain. PRF, as a neuromodulation technique, does not induce tissue damage. It’s benefits—safety, minimal invasiveness, repeatability, and prolonged analgesic effects—have made it a widely used therapy for various pain syndromes [7], including CEH [8].

Historically, C2 DRG puncture has been performed using X-ray fluoroscopy [9, 10, 11] or computed tomography (CT) guidance [12]. However, this approach presents several challenges. The inability to directly visualize the C2 DRG under fluoroscopy requires reliance on indirect bony landmarks (such as the atlantoaxial joints) for localization, which can reduce puncture accuracy. Furthermore, the C2 DRG is located adjacent to critical tissues including the spinal cord, cerebrospinal fluid, dural sacs, and blood vessels. These structures are difficult to distinguish under fluoroscopic guidance, increasing the risk of inadvertent puncture.

In recent years, significant advancements have been made in ultrasound-guided puncture technologies. The use of ultrasound imaging has enabled clear visualization of the C2 DRG and its adjacent structures, improving real-time accuracy, safety, and efficacy of the procedure [13, 14]. This approach shows significant promise for future applications; however, the number of related studies remains limited. Michael D. *et al*. [15] used ultrasound imaging of the C1–2 interspace in the adult cervical spine to provide a clear visual representation of the spinal cord, C2 nerve, and C2 DRG. These findings suggest that this approach may be utilized safely and effectively to facilitate C2 DRG puncture. The present study introduced an innovative ultrasound-guided approach to C2 DRG puncture and evaluated the effectiveness and safety of ultrasound-guided C2 DRG pulsed-radiofrequency (PRF) treatment for CEH within the C1–2 interspace in a retrospective clinical cohort.

## 2. Patients and methods

### 2.1 Criteria for patient selection

This retrospective cohort study gathered data from January 2023 to July 2023, focusing on patients with unilateral CEH who had complete clinical records.

Inclusion criteria:

a. Patients were diagnosed with unilateral CEH according to the International Classification of Headache Disorders, 3rd Edition (ICHD-3) [1] and the Cervicogenic Headache International Study Group (CHISG) criteria [16] following a comprehensive history and physical examination (Tables 1,2).

**Table 1.** IHS diagnostic criteria (3rd edition, 2018).

Criterion	Description
A	Any headache fulfilling criterion C
B	Clinical and/or imaging evidence of a disorder or lesion within the cervical spine or soft tissues of the neck, known to be able to cause headache
C	Evidence of causation demonstrated by at least two of the following:
	1. headache has developed in temporal relation to the onset of the cervical disorder or appearance of the lesion
	2. headache has significantly improved or resolved in parallel with improvement in or resolution of the cervical disorder or lesion
	3. cervical range of motion is reduced and headache is made significantly worse by provocative maneuvers
	4. headache is abolished following diagnostic blockade of a cervical structure or its nerve supply
D	Not better accounted for by another ICHD-3 diagnosis

*Notes: IHS, International Headache Society; ICHD-3: International Classification of Headache Disorders, 3rd Edition.*

**Table 2.** CHISG major diagnostic criteria.

Criterion	Description
I	Symptoms and signs of neck involvement:
	(a) Precipitation of head pain, similar to the usually occurring one: (1) by neck movement and/or sustained awkward head positioning; (2) by external pressure over the upper cervical or occipital region on the symptomatic side.
	(b) Restriction of the range of motion in the neck.
	(c) Ipsilateral neck, shoulder, or arm pain of a vague, non-radicular nature or, occasionally, radicular arm pain.
II	Confirmatory evidence by diagnostic anesthetic blockades
III	Unilaterality of the pain, without side shift

*Notes: CHISG, Cervicogenic Headache International Study Group.*

b. Headache meeting the following criteria: (1) Unilateral pain originating in the cervical region and radiating to the head-occipital or temporal areas; (2) Headache triggered by neck movement or prolonged uncomfortable head postures; (3) Headache was accompanied by simultaneous cervical discomfort.

c. Patients aged between 18 and 70 years.

d. Patients who experienced headache symptoms for at least 3 months.

e. Patients who provided informed consent and agreed to undergo the designated therapy.

Exclusion criteria:

a. Patients with malignancies or a recent or past history of head and neck trauma.

b. Patients with comorbidities that may cause headache symptoms.

c. Patients with a history of previous invasive therapies for CEH.

d. Patients diagnosed with diabetes mellitus, rheumatoid arthritis, or coagulation disorders.

Setting: This study was conducted in the pain management department within the inpatient unit of our hospital.

### 2.2 Technique

Upon entering the treatment room, all patients were positioned in a lateral recumbent posture with the afflicted side facing upward, and their heart rate, electrocardiogram, noninvasive blood pressure, and pulse oxygen saturation were continuously monitored. Oxygen inhalation via a low-flow (2 L/min) nasal cannula was provided. A curved array probe (2–5 MHz, Wisonic Navi, Shenzhen, China) was employed to establish a depth to 6–10 cm. The probe was positioned approximately under the occipital bone in a sagittal orientation down the spine, confirming the posterior arch of the atlas, the lamina of the axis, and the lamina of C3 by ultrasound. The C1–2 gap was positioned centrally in the ultrasound picture, and the ultrasound probe was rotated 90°. At this angle, the ultrasound probe was positioned horizontally on the spine, clearly displaying the cervical spinal cord, the C2 DRG, and the lateral aspect of the C2 vertebral body. The location for the ultrasound-guided puncture was then marked. Following skin disinfection, the ultrasonic probe was encased in a sterile protective sheath and repositioned. In Doppler color mode, the image depicted the C1–2 laminar area in horizontal alignment, showing the vertebral artery and surrounding blood vessels. Local infiltration anesthesia was administered using 3 mL of 1% lidocaine on the skin of the dorsal spine, which was positioned 1 cm beside the probe. A disposable radiofrequency cannulated puncture needle (10 cm, 22 G, with a 5 mm exposed tip) was used to carefully puncture the C2 DRG in-plane, meticulously avoiding blood vessels and the spinal cord under ultrasound guidance (Figs. 1,2).

**Fig. 1.** Ultrasound probe placement and corresponding ultrasound images. (A) Anatomical schematic illustrating the placement of ultrasound probe. (B) Ultrasound image showing the probe in a sagittal position with the suboccipital bone. (C) Color Doppler ultrasound image at the C1–2 level. (D) Ultrasound image of the C2 DRG puncture. (OB, occipital bone; OCI, obliquus capitis inferior muscle; C1, the posterior arch of atlas; C2, lamina of axis; C3, lamina of C3; C2 LB, lateral block of C2 vertebral body; C2 SP, spinous process of C2; Red arrow, vertebral artery; Red dotted line, cervical spinal cord; White arrow, needle; Green arrow, needle tip; Yellow arrow, C2 DRG; Yellow triangular arrow, anterior epidural space.)

**Fig. 2.** Ultrasound-Guided C2 DRG Puncture and Vessel Visualization. (A) Patient’s position and performance of the C2 DRG puncture with needle in-plane approach. (B) Color Doppler ultrasound image showing vessels around C2 DRG. (C2 LB, lateral block of C2 vertebral body; Yellow arrow, C2 DRG; Red arrow, vertebral artery; Green triangular hollow arrow, blood vessels around C2 DRG; Yellow triangular arrow, anterior epidural space.)

Upon puncture completion puncture, the radiofrequency needle core was inserted, and sensory stimulation was delivered at a voltage <0.3 V and a frequency of 50 Hz to elicit posterior occipital discomfort, confirming accurate placement. Once the target location was verified, radiofrequency pulse neuromodulation was initiated. Treatment parameters included an electrode tip temperature of 42 °C, with a pulse frequency of 2 Hz, a pulse width of 20 ms, an output voltage of 45 V, and a duration of 600 s. Following radiofrequency treatment, 2 mL of 0.9% normal saline along with 2 mg of dexamethasone was injected via the puncture needle. The patient was then required to remain in bed for 2 hours post-procedures.

### 2.3 Clinical evaluation

#### 2.3.1 Headache intensity

The intensity of headaches was evaluated before therapy and subsequently on the day following treatment, the third day, and at weeks 1, 2, 4, 8, 12, 16, 20 and 24 post-treatments with the Numerical Rating Scale (NRS) for pain. The NRS is a pain assessment instrument that uses a scale from 0 to 10 to measure the intensity of pain experienced by the patient, where 0 indicates no pain, and 10 represents the most severe pain imaginable. The number of patients who achieved a 70% reduction in NRS scores (considered significantly effective) or a 50% reduction (considered successful) at weeks 4, 12 and 24 post-treatment was compared with pre-treatment levels.

#### 2.3.2 Neck disability index

Furthermore, patients’ functionality was evaluated with the neck disability index (NDI) before and during cervical spine intervention. The NDI assesses 10 domains, namely, pain severity, personal care, weight lifting, reading, headache, attention, work, driving, sleep, and recreation, with scores ranging from 0 to 5 for each category. The total scores ranges from 0 (no disability) to 50 (complete disability). Evaluations were performed before therapy and at 4, 12 and 24 weeks following treatment.

#### 2.3.3 Ultrasound imaging and negative reactions

The ultrasound images are captured and analyzed to identify vessels surrounding the C2 DRG and along the puncture path throughout the therapy process. Adverse reactions, including nerve, blood vessel and spinal cord damage, along with symptoms such as unconsciousness, dizziness, nausea and palpitation, were documented.

### 2.4 Statistical analysis

Statistical analysis was conducted utilizing SPSS 27.0 statistical software (SPSS Inc., Chicago, IL, USA). All the data were subjected to the Shapiro-Wilk test to assess normality before analysis, with continuous data presented as the mean ± standard deviation (x̄ ± SD) or median (interquartile range (IQR). Comparisons were made between pre- and post-treatment using repeated-measures Analysis of variance (ANOVA) or nonparametric tests as appropriate. Categorical data were expressed as percentages and analyzed using chi-square tests or Fisher’s exact tests. A *p*-value of < 0.05 was considered statistically significant.

## 3. Results

### 3.1 Characteristics of the included patients

The study included 29 patients, comprising 13 men and 16 women, with an average age of 51.34 ± 9.97 years (range: 35–66 years), weight of 61.83 ± 7.9 kg, height of 1.67 ± 0.07 m, and Body mass index (BMI) of 22.13 ± 2.04 kg/m^2^. The mean duration of headaches was 26.90 ± 11.70 months, with 15 patients experiencing right-sided headaches and 14 patients experiencing left-sided headaches (Table 3).

**Table 3.** Basic characteristics of patients (N = 29).

Variables		Values
Age (years)		51.34 ± 9.97
Sex, n (%)		
	Female	16 (55.17)
	Male	13 (44.83)
Duration (months)		26.90 ± 11.70
BMI (kg/m^2^)		22.13 ± 2.04
Side of symptoms, n (%)		
	Right	15 (51.72)
	Left	14 (48.28)

*Notes: BMI, Body Mass Index.*

### 3.2 Pain assessment

Patients exhibited substantial improvements in headache symptoms at all post-treatment intervals relative to pre-treatment intervals (*p* < 0.05) (Table 4). However, patients 4 and 9 maintained the same headache severity at 24 weeks as before treatment. Patients whose NRS score was 70% lower than baseline (significantly effective) and those whose scores were 50% lower (effective) at weeks 4, 12 and 24 post-treatments underwent statistical analysis (Table 5).

**Table 4.** NRS scores at each time point for patients (N = 29).

Time Point	Median	Range (IQR)
Before Treatment	7	(6.5, 8.0)
On the day of treatment	0	(0.0, 1.0)*
3 Days Post-treatment	3	(2.0, 3.0)*
1 Week Post-treatment	3	(2.0, 3.0)*
2 Weeks Post-treatment	3	(2.0, 4.0)*
4 Weeks Post-treatment	3	(2.0, 4.0)*
8 Weeks Post-treatment	3	(2.0, 4.0)*
12 Weeks Post-treatment	3	(2.0, 4.0)*
16 Weeks Post-treatment	3	(3.0, 4.0)*
20 Weeks Post-treatment	3	(2.5, 4.0)*
24 Weeks Post-treatment	3	(3.0, 4.5)*

Notes: *indicates a significant decrease in NRS score compared to pre-treatment; NRS, Numerical Rating Scale; IQR, Interquartile Range.

**Table 5.** Headache relief in patients at 4, 12 and 24 weeks.

Time Point	≥70% Reduction, n (%)	≥50% Reduction, n (%)	<50% Reduction, n (%)
4 Weeks Post-treatment	4 (13.79)	21 (72.41)	8 (27.59)
12 Weeks Post-treatment	5 (17.24)	20 (68.97)	9 (31.03)
24 Weeks Post-treatment	5 (17.24)	20 (68.97)	9 (31.03)

### 3.3 NDI scores

The pre-treatment NDI was 35.21 ± 6.72, while the post-treatment NDI scores were 16.79 ± 5.94 at week 4, 17.9 ± 6.17 at week 12, and 18.52 ± 8.04 at week 24. The post-treatment NDI scores at weeks 4, 12 and 24 showed significant improvements (*p* < 0.05) compared to the pre-treatment NDI score (Table 6).

**Table 6.** Patients’ NDIs at 4, 12 and 24 weeks (N = 29).

Time Point	NDI (Mean ± SD)
Before Treatment	35.21 ± 6.72
4 Weeks Post-treatment	16.79 ± 5.94*
12 Weeks Post-treatment	17.90 ± 6.17*
24 Weeks Post-treatment	18.52 ± 8.04*

Notes: *indicates a significant decrease in NDI compared to pre-treatment. NDI, Neck Disability Index; SD, standard deviation.

### 3.4 Abnormal vessels

Ultrasound imaging during therapy revealed blood vessels adjacent to the C2 DRG and the lateral block of the C2 vertebral body in four patients (13.8%) (Fig. 2).

### 3.5 Adverse reactions

None of the 29 patients experienced severe problems, including neurological, vascular, or spinal cord injuries, dizziness, nausea, palpitation. However, three patients (10.3%) reported noticeable pain at the puncture site post-treatment, which was successfully mitigated by administering 0.1 g of celecoxib.

## 4. Discussion

This was a retrospective cohort study with 29 patients with CEH. Among the 29 patients treated, 27 reported substantial alleviation of headache symptoms. Specifically, 17.24% of the patients attained a substantial effectiveness rate (with NRS ratings decreasing by 70% or more), whereas 68.97% achieved an efficacy rate (with NRS scores decreasing by 50% or more). Furthermore, patients exhibited significant improvements in their NDI scores. No participants experienced significant side effects from the treatment, indicating that ultrasound-guided C2 DRG PRF therapy at the C1–2 level is likely a safe and effective choice for treating CEH.

In our investigation, the treatment effectiveness rate at 6 months was at least comparable to that suggested by X-ray techniques [9, 11] and fewer side effects were observed. This result may be attributed to three possible factors. First, hazardous structures are identifiable using ultrasonography, facilitating proximity to the C2 DRG, and, therefore, increasing the efficacy rate. Second, the targets differ with one directed at an indirect marker (the atlantoaxial joints) and the other toward a direct target (the C2 DRG). Third, the use of water-soluble hormones may help eradicate localized aseptic inflammation and edema.

Recent research has explored ultrasound-guided therapeutic approaches for C2 DRG due to advancements in ultrasound technology. Baishan Wu *et al*. [13] conducted ultrasound-guided puncture of the C2 DRG, positioned deep to the obliquus capitis inferior (OCI), for coblation therapy in 26 patients, achieving effectiveness rates of 92.31% and 88.46% at the 12-week and 24-week follow-ups, respectively. Lu Hua *et al*. [14] employed the same technique of ultrasound-guided puncture on the C2 DRG for PRF in conjunction with an ultrasound-guided stellate ganglion block, resulting in a reduction in the visual analogue scale (VAS) pain score from 6.53 ± 1.17 before treatment to 0.97 ± 0.76 at the 6-month of follow-up.

Our treatment was less successful (68.97% *vs.* 88.46%) than ultrasound-guided C2 DRG procedures targeting the obliquus capitis inferior (OCI) [13, 14]. This gap may be attributed to two potential factors. Initially, PRF alone neuromodulates the C2 DRG, whereas coblation therapy obliterates it, thereby impairing nerve transmission and yielding superior therapeutic outcomes. Second, the integration of PRF therapy targeting the C2 DRG with other invasive interventions may augment the therapeutic efficacy of CEH.

PRF is a neuromodulation treatment that preserves nerves and adjacent tissues. We chose PRF because ultrasound imaging revealed that the C2 DRG is in proximity to critical tissues, including the spinal cord and vertebral artery, which may sustain injury if radiofrequency ablation (RFA) is conducted. Compared with RFA, PRF may have a diminished therapeutic impact or a reduced duration of pain alleviation. Hamer *et al*. [17] included 40 patients who underwent RFA to induce thermal injury to the C2 DRG, hence disrupting nerve transmission to mitigate headache symptoms. At 6 months of follow-up, 35% of patients experienced complete remission from pain symptoms, while 70% achieved above 80% alleviation, and the incidence of related problems reached 13%. Several studies have administered long-acting granular glucocorticoids and local anesthetics around the C2 nerve root for the treatment of CEH [10, 18]. However, owing to the proximity of the puncture needle tip to the spinal cord and blood vessels in our investigation, only dexamethasone (a water-soluble hormone) and saline were injected through the puncture needle post-PRF to mitigate risk.

While no significant concerns related to the C2 DRG have been documented with fluoroscopic and ultrasound guidance, four patients (13.8%) presented with abnormal vessels around the C2 DRG during our procedure (Fig. 2). Ultrasound guidance facilitated the avoidance of aberrant blood vessels during the real-time puncture procedure and enabled the observation of drug diffusion, thus increasing operational safety.

Our research has several limitations. Firstly, we conducted a single-center retrospective analysis that included a limited cohort of patients. Future studies should encompass more comprehensive clinical trials to investigate the efficacy of this therapeutic methodology. Secondly, several patients opted for additional therapies, including physical therapy, manipulation, and oral medicine, after radiofrequency treatment. The omission of these supplementary therapies in this study slightly undermined its clinical validity. Subsequent research should incorporate these factors to facilitate a more thorough assessment. Thirdly, this trial did not provide the mean NRS scores for headache severity at different follow-up intervals before and after therapy. Furthermore, we did not collect data about the frequency and duration of headache episodes. Incorporating such indicators in future studies may enhance the evaluation of therapeutic efficacy. Multicenter, large-sample, double-blind, randomized clinical studies are essential to overcome these constraints and corroborate our findings.

## 5. Conclusions

This single-center retrospective cohort study demonstrated that ultrasound-guided C2 DRG PRF, in conjunction with the injection of water-soluble hormones at the C1–2 level, may provide a safer and more effective alternative for treating CEH compared to fluoroscopic methods. However, due to the limitations of this retrospective investigation, further research through multicenter, randomized controlled trials is essential to validate these findings and to further ascertain the efficacy and safety of this methodology.
